# Characterizing Fatigue-Related White Matter Changes in MS: A Proton Magnetic Resonance Spectroscopy Study

**DOI:** 10.3390/brainsci9050122

**Published:** 2019-05-27

**Authors:** Kalyan Yarraguntla, Fen Bao, Samuel Lichtman-Mikol, Sara Razmjou, Carla Santiago-Martinez, Navid Seraji-Bozorgzad, Shitiz Sriwastava, Evanthia Bernitsas

**Affiliations:** 1 The Sastry Foundation Advanced Imaging Laboratory, Wayne State School of Medicine, 4201 St Antoine, Detroit, MI 48201, USA; kalyancy@wayne.edu (K.Y.); fbao@med.wayne.edu (F.B.); slichtma@med.wayne.edu (S.L.-M.); srazmjou@med.wayne.edu (S.R.); nseraji@wayne.edu (N.S.-B.); 2 Multiple Sclerosis Center, Department of Neurology, Wayne State School of Medicine, 4201 St Antoine, 8C-UHC Detroit, MI 48201, USA; du2067@wayne.edu (C.S.-M.); ssriwast@med.wayne.edu (S.S.)

**Keywords:** fatigue, multiple sclerosis, magnetic resonance spectroscopy, N-acetylaspartate, N-acetylaspartylglutamate

## Abstract

Few cross-sectional studies have investigated the correlation between neurochemical changes and multiple sclerosis (MS) fatigue, but little is known on the fatigue-related white matter differences between time points. We aim to investigate the longitudinal neurometabolite profile of white matter in MS fatigue. Forty-eight relapsing remitting multiple sclerosis (RRMS) patients with an expanded disability status scale (EDSS) ≤ 4 underwent high field ^1^H-multivoxel magnetic resonance spectroscopy (MRS) at baseline and year 1. Fatigue severity was evaluated by the fatigue severity scale (FSS). Patients were divided into low (LF, FSS ≤ 3), moderate (MF, FSS = 3.1–5), and high fatigue (HF, FSS ≥ 5.1) groups. In a two-way analysis of variance (ANOVA), we observed a decline in the ratio of the sum of N-acetylaspartate (NAA) and N-acetylaspartylglutamate (NAAG) to the sum of creatine (Cr) and phosphocreatine (PCr) in the right anterior quadrant (RAQ) and left anterior quadrant (LAQ) of the MRS grid in the HF group at baseline and year 1. This decline was significant when compared with the LF group (*p* = 0.018 and 0.020). In a one-way ANOVA, the fatigue group effect was significant and the ratio difference in the right posterior quadrant (RPQ) and left posterior quadrant (LPQ) of the HF group was also significant (*p* = 0.012 and 0.04). Neurochemical changes in the bilateral frontal white matter and possibly parietooccipital areas were noted in the HF group at two different time points. Our findings may shed some light on the pathology of MS fatigue.

## 1. Introduction

Fatigue is one of the most prominent and disabling symptoms, affecting up to 80% of multiple sclerosis (MS) patients [1]. It is defined as difficulty initiating or sustaining voluntary activities and a feeling that a given activity requires disproportionate effort [2,3]. It has a tremendous effect on the quality of life, employment status, and patient’s disability [4]. Previous cross-sectional studies have reported the involvement of the thalamus, basal ganglia, superior cerebellar peduncle, and right temporal cortex that correlate with the fatigue severity scale in MS [5,6]. Few studies have reported fronto-parietal tract disruption and extensive white matter lesions in highly fatigued patients [7,8]. There is a paucity in the literature of imaging studies examining the longitudinal nature of MS. One study reported an association between fatigue severity variation and the disruption of neuronal architecture in the right temporal cortex [9], and another study demonstrated that thalamic and cerebellar atrophy may be predictors of subsequent fatigue development [10]. There are even fewer cross-sectional studies investigating an association between neurometabolite changes in white matter and MS fatigue. Neurochemical alterations in the pons, hypothalamus, lentiform nucleus, gray matter, and diffuse axonal injury have also been reported to be associated with MS fatigue pathology [11,12,13,14,15]. Despite extensive research, the pathophysiology and longitudinal evolution of fatigue symptoms in the white matter of MS patients is complex and remains unclear.

Proton magnetic resonance spectroscopy (^1^H-MRS) is a non-invasive imaging technique that has been utilized to evaluate neuronal health by detecting subtle biochemical changes in both lesions and normal-appearing white matter (NAWM) [16]. The metabolites determined by this technique are the N-acetyl aspartate (NAA) and N-acetylaspartylglutamate (NAAG), which are synthetized in neuronal mitochondria and their ratio with creatinine (Cr) and phosphocreatinine (PCr) are reported to be markers of neuronal and axonal integrity [17,18,19,20]. In a small study, Zaini et al. demonstrated lower NAA/Cr in the tegmental pons of a high fatigue (HF) MS population compared with healthy controls [11]. Tellez et al. reported a significant decrease in NAA/Cr in the lentiform nucleus in MS patients with fatigue, while Kantorova et al. reported hypothalamic metabolic alterations in MS fatigue [12,13]. In a well-designed study, Pokryszko-Dragan et al. examined both gray and white matter and found reduced NAA/Cr levels in the posterior cingulate gyrus and parietal white matter in MS patients with fatigue compared with healthy controls; however, no significant relationship was found between magnetic resonance spectroscopy (MRS) parameters and fatigue score [14]. In a retrospective study, including both relapsing remitting multiple sclerosis (RRMS) and progressive MS patients with low disability, Tartaglia et al. reported a reduced NAA/Cr ratio in the HF group compared with the low fatigue (LF) group, and a significant linear correlation between NAA/Cr and Fatigue Severity Scale (FSS) scores across all patients after correcting for the Expanded Disability Status Scale (EDSS), disease duration, and T2 lesion load [15]. In most studies, the focus was either on specific regions of the brain or on the whole brain, which may limit the potential to trace fatigue pathways and understand the evolution of associated pathology. In MS, where the entire white matter (WM) is susceptible to demyelination, the contribution of the occipitoparietal WM in MS fatigue has not been investigated. 

The spectroscopy technique has been utilized in MS, and it was reported that the NAA/Cr ratio correlates strongly with EDSS in early MS, with this correlation being stronger in patients with mild disability rather than in those with severe disability [21,22]. Decreased NAA and Cr combined with higher levels of choline (Cho) compared with controls were found in the NAWM of MS patients [23]. Additionally, hypointense T1 lesions showed a lower concentration of NAA and Cr compared with NAWM [24]. Previous studies have also shown that increased Cho and Cr levels are present during formation of pathologically “mild” lesions and high levels of glutamate (Glu) herald the appearance of new T2 visible WM lesions [25]. 

In view of the subjective nature of fatigue and the sensitivity of the spectroscopy technique to record subtle neurometabolite changes, using high field ^1^H-MRS, we aimed to measure the chemical alterations in white matter (WM) that may have a role in fatigue pathology. We examined the changes in the (NAA+NAAG)/(Cr+PCr) ratio in a multi-voxel MRS grid that encompasses frontoparietal and occipitoparietal regions along with the central region of the corpus callosum in RRMS patients who reported fatigue, over a period of 1 year.

The goal of this study was to compare changes in the neurometabolite profile between MS patients with different levels of fatigue at two different time points, thus enhancing our understanding of MS fatigue in highly fatigued MS patients. 

## 2. Methods

### 2.1. Patient Recruitment and Selection Criteria

Forty-eight relapsing remitting multiple sclerosis patients (RRMS) diagnosed per the revised 2010 McDonald criteria were recruited from the MS clinic and enrolled in this observational longitudinal study [26]. We included patients between 18 and 55 years who denied sleep disorders and other causes of fatigue such as active infection, malignancy, anemia, thyroid, or adrenal disease. All included patients had an EDSS ≤ 4 in order to minimize the effect of physical disability on fatigue [27]. We excluded patients who were pregnant or had other neurological or psychiatric disorders, such as depression or anxiety, because of these disorders’ established association with fatigue [28,29]. Patients who had a relapse or received steroids within a period of 3 months prior to study enrollment were excluded. Moreover, patients on antidepressants, psychoactive medications, stimulants, or medications for the symptomatic treatment of fatigue were also excluded. On the same day of MRS acquisition at baseline and year 1, participants underwent a neurological evaluation, including EDSS. Fatigue severity was assessed at baseline and year 1 using the Fatigue Severity Scale (FSS), given that it is shorter than the Modified Fatigue Impact Scale (MFIS) and has high test-retest consistency (Cronbach’s alpha value = 0.89 or higher) [30,31,32,33]. FSS is a self-reported questionnaire consisting of nine statements with a seven-point scale response per statement, with lower scores indicating less fatigue. The MS patients with a mean FSS score ≥ 5.1 were categorized as high fatigued (HF), those with a mean FSS score ≤ 3 as low fatigued (LF), and those with an FSS score between 3.1–5 were classified as moderately fatigued (MF). The effect of disease-modifying agents was minimal, as all the patients included in this study were on fingolimod.

The institutional review board approved the study protocol (ethic code: # 034815MP4F). Written informed consent was obtained from all research subjects.

### 2.2. Image Acquisition

**Figure 1:**
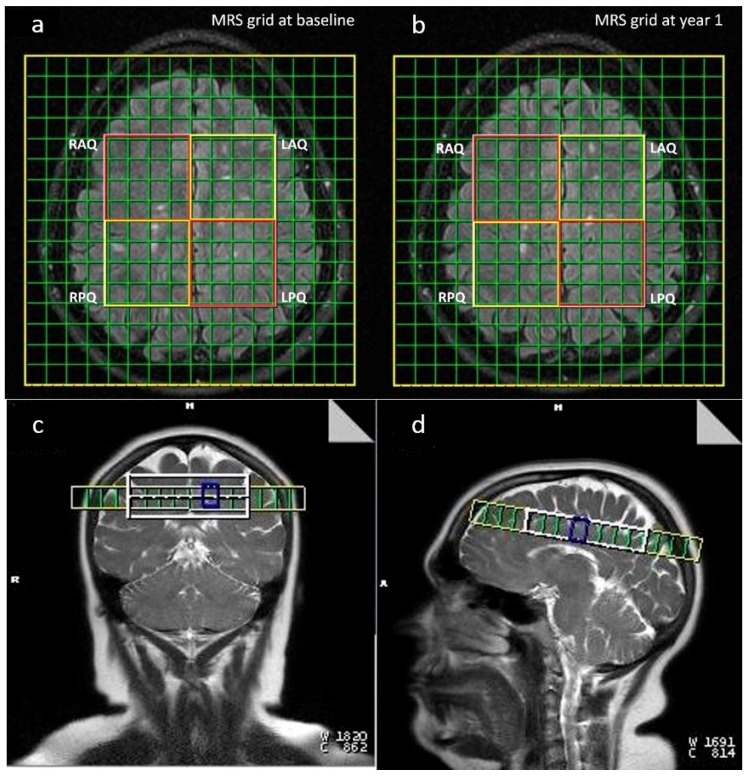
Axial View showing magnetic resonance spectroscopy (MRS) grid placed at same level at baseline and year 1 (**a**,**b**). Coronal and sagittal view of the MRS grid location in the periventricular area (**c**,**d**). RAQ—Right Anterior Quadrant, LAQ—Left Anterior Quadrant, RPQ—Right Posterior Quadrant, LPQ—Left Posterior Quadrant.

A two-dimensional (2D) Multivoxel point resolved spectroscopy (PRESS) sequence was performed on a Siemens 3T Verio MR scanner (Siemens Healthineers, Munich, Germany) by using a 12 channel radio frequency head coil to estimate the sum of NAA and NAAG relative to the sum of Cr and phosphocreatine (PCr) concentrations (NAA+NAAG)/(Cr+PCr). The multi-voxel slab was placed immediately rostral to the lateral ventricles focusing on a large area of central white matter. This slab was manually placed in the position that is parallel to a line joining the inferior parts of corpus callosum at both time points as shown in Figure 1c,d. The lower border of central voxel was placed immediately above the central portion of body of corpus callosum. At the same time, the lower border of the anterior voxel of the slab was matched with the anterior border of the brain in the frontal area. It was optimal to void inclusion of CSF (cerebrospinal fluid) from the lateral ventricles although in some patients, but this may be unavoidable due to individual brain and skull size variation. On the subsequent MRS at year 1, the same process was repeated. Time repetition to echo time (TR/TE) (milliseconds) = 1500/135, chemical shift imaging (CSI) matric size = 16 × 16 voxels and voxel size 10 × 10 × 15 millimeters (1.5 mL), voxel of interest (VOI) = 8 × 8 voxels, number of averages = 8 and number of measurements = 1, water suppression band width = 50 Hz and acquisition time = 11 min and 30 s.

A T2-weighted FLAIR (fluid-attenuated inversion recovery) image (TR/TE = 9000/128 milliseconds, inversion time = 2500 milliseconds, flip angle = 150°, acquisition matrix size = 256 × 192, voxel size = 1 × 1 × 3 mm, 46 contiguous axial slices) and a T2-weighted turbo spin-echo image (TR/TE = 7810/97 milliseconds, flip angle = 120°, acquisition matrix size = 640 × 480, voxel size = 0.4 × 0.4 × 3 mm, 46 contiguous axial slices) were acquired and covered the whole brain to measure the lesion volume (Figure 1).

### 2.3. Image Processing

**Figure 2:**
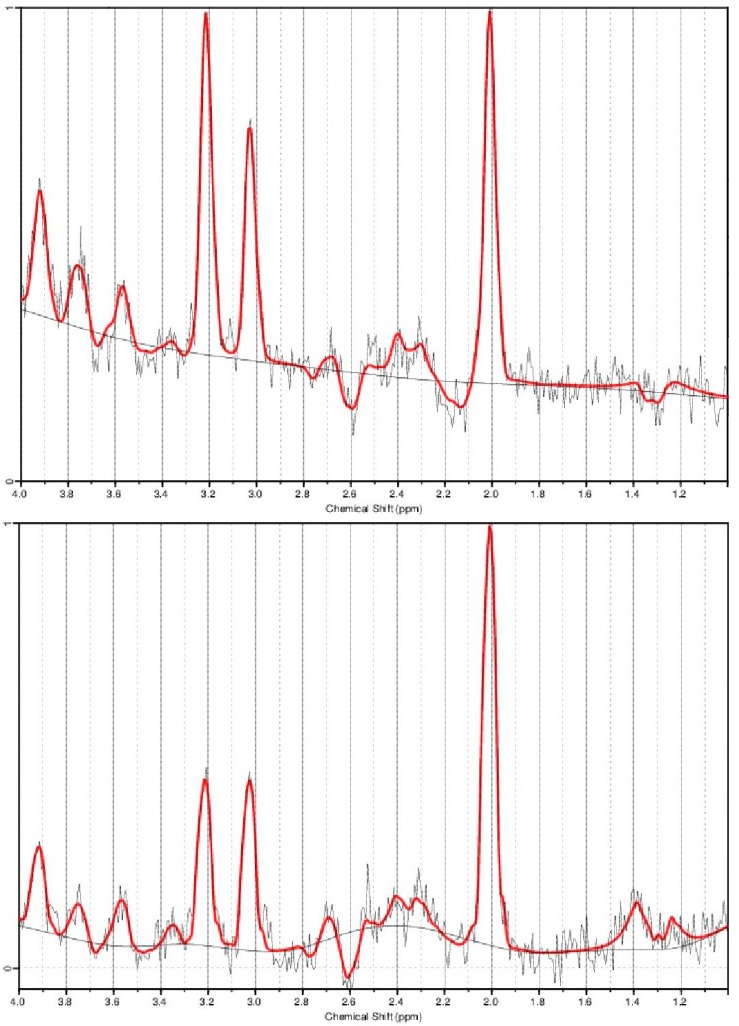
Sample spectra of high fatigue (HF) (above) and low fatigue (LF) (below). The tallest peak represents N-acetylaspartate (NAA)+N-acetylaspartylglutamate (NAAG). The second tallest peak close to NAA+NAAG peak represents creatinine (Cr)+phosphocreatine (PCr).

Multi-voxel 1H spectroscopy images were analyzed by using *LCModel* software (Version 6.2, LCModel Inc., Oakville, ON, Canada) (http://s-provencher.com/lcmodel.shtml) to estimate the (NAA+NAAG)/(Cr+PCr) concentration ratio in 8 × 8 voxels of interest (VOI) in the central WM as shown in Figure 2. *LCModel* uses a custom-made basis set to estimate the relative metabolite concentration and Cramer–Rao bound statistic to determine the confidence interval of the estimates [34]. Spectra with a Cramer–Rao bound greater than 20% were excluded from the analysis as previously performed [35]. Overall, no voxels were excluded at each time point.

The 64 voxels were divided into four quadrants: left anterior, left posterior, right anterior, and right posterior, with each quadrant consisting of 16 voxels as shown in Figure 1a,b. The anterior quadrants (4 × 4) comprise of frontoparietal WM tracts and posterior quadrants comprise of parieto-occipital WM tracts. The voxels with metabolite concentration ratios with a standard deviation higher than 20% were excluded from the analysis, thereby minimizing the effect of gray matter and non-brain tissue on the concentration ratio. In each quadrant, the average of (NAA+NAAG)/(Cr+PCr) ratios of all valid voxels was calculated and used for the statistical analysis [36,37,38].

The dispersion of NAA, choline, and creatine peaks is presented in the sample spectra of a single voxel (Figure 2).

### 2.4. Lesion Volume Measurement

We identified and outlined T2 lesions on T2 images with reference to FLAIR images by the semi-automated edge detection contouring/thresholding technique, and then calculated the T2 lesion volumes on T2 images. We used the DispImage image analysis package V4.9 (Dave Plummer, University College London Hospitals NHS Trust, London, UK) for lesion analysis [39].

### 2.5. Statistics

As previously mentioned, our MS patients were categorized based on a fatigue score into LF, MF, and HF groups. A chi-square test was performed to assess the variation in sample size of gender and ethnicity between the groups. The variation in age, EDSS, disease duration, and lesion load between the three fatigue groups were evaluated using univariate analysis and the *p*-value was reported. With spectroscopy data, we initially performed boxplot analyses of the mean (NAA+NAAG)/(Cr+PCr) ratio within each quadrant of the MRS grid to detect the outliers beyond the 95th percentile in each fatigue group. No outliers were identified. Then, we performed a two-way analysis of variance (ANOVA) applying pairwise Bonferroni post-hoc testing with the (NAA+NAAG)/(Cr+PCr) ratio of each quadrant as a dependent variable and the fatigue group and time point as independent variables to explore the main effects, interactions, and pairwise comparisons of the neurometabolite ratio. Likewise, the variation in the (NAA+NAAG)/(Cr+PCr) ratio difference in each quadrant was also assessed between the three fatigue groups using a one-way ANOVA. Bonferroni post-hoc analysis was performed to report the pairwise comparisons. The *p* < 0.05 was considered statistically significant. All results are expressed as mean ± standard error of mean (SEM) along with maximum and minimum range. Statistical analysis was performed using the Statistical Package for the Social Sciences (IBM Corp. released 2017. IBM SPSS Statistics for Windows, Version 25.0. Armonk, NY, USA).

## 3. Results

### 3.1. Patient Demographics

Forty-eight patients with RRMS participated in this observational study. Our sample consisted of 33 women with a mean age of 41 (± 2.4) years and 15 men with a mean age of 39 (± 2.3) years. Patients’ demographic and clinical characteristics in the total sample and within each group are presented in Table 1. As shown in Table 1, we did not observe a significant difference between the fatigue groups in age, disease duration, T2 lesion load, and EDSS score in our univariate analysis. Furthermore, we did not observe a significant variation in FSS score, EDSS score, or lesion load between the baseline and year 1 within each group in our paired sample *t*-test. The variation in sample size of gender and ethnicity within each group was not significant in our chi-square test.

### 3.2. Spectroscopy Findings

**Figure 3:**
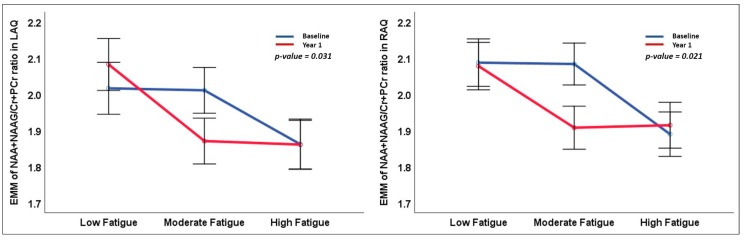
Line graphs represent the estimated marginal mean and standard error of mean (SEM) of the NAA+NAAG/Cr+PCr ratio in the right anterior quadrant (RAQ) and the left anterior quadrant (LAQ) between the fatigue groups at baseline and year 1. The *p*-value represents the statistical significance of the main effect of fatigue groups in the two-way ANOVA.

**Figure 4:**
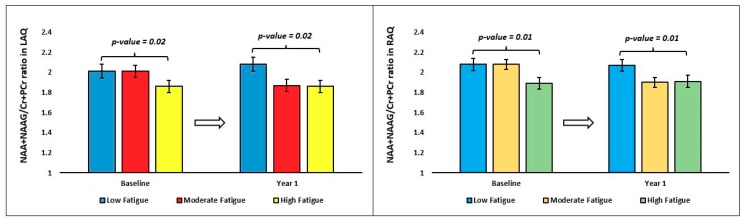
Bar graphs representing the mean and standard error of mean (SEM) of NAA+NAAG/Cr+PCr ratio in right anterior quadrant (RAQ) and left anterior quadrant (LAQ) between the fatigue groups at baseline and year 1. The *p*-value represents the statistical significance of pairwise comparisons in the two-way ANOVA.

Two-way (2 × 3) ANOVAs revealed significant main effects of the factor group for the right anterior quadrant (RAQ) (F(2, 88) = 3.99, *p* = 0.022) and the left anterior quadrant (LAQ) (F(2, 89) = 3.6, *p* = 0.031) (Table 2 and Figure 3). Within the RAQ, the (NAA+NAAG)/(Cr+PCr) ratio was significantly higher in the LF group compared with the HF group (*p* = 0.018) in our Bonferroni post-hoc testing. Similarly, the (NAA+NAAG)/(Cr+PCr) ratio of the LAQ was significantly higher in the LF group compared with the HF group (*p* = 0.02) (Figure 4).

**Figure 5:**
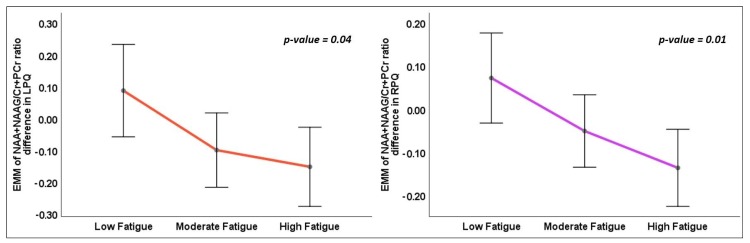
Line graphs representing the estimated marginal mean and standard error of mean (SEM) of the NAA+NAAG/Cr+PCr ratio difference (year 1 minus baseline) in the left posterior quadrant (LPQ) and right posterior quadrant (RPQ) between the fatigue groups at baseline and year 1. The p-value represents the statistical significance of the main effect of fatigue groups in the one-way ANOVA.

**Figure 6:**
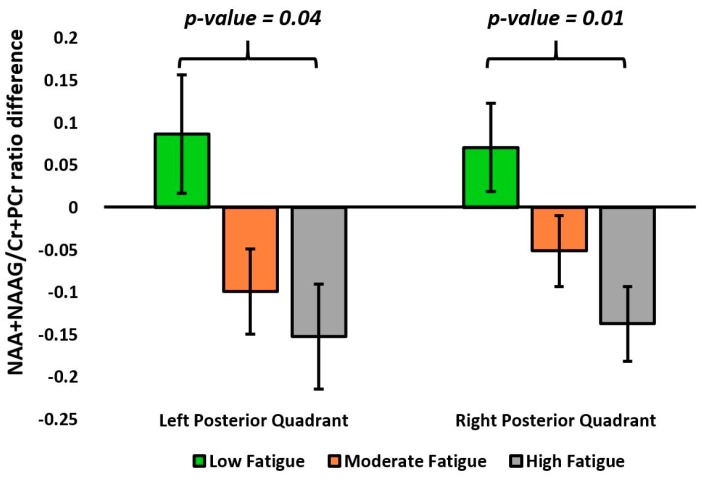
Bar graphs representing the mean and standard error of mean (SEM) of the NAA+NAAG/Cr+PCr ratio difference (year 1 minus baseline) in the left posterior quadrant (LPQ) and right anterior quadrant (RPQ) between the fatigue groups. The *p*-value represents the statistical significance of pairwise comparisons in the one-way ANOVA.

In the one-way ANOVA, the (NAA+NAAG)/(Cr+PCr) ratio difference between the time points showed significant variation within the right (RPQ, main effect *p* = 0.015) and left (LPQ, main effect *p* = 0.041) posterior quadrants between the fatigue groups (Table 3 and Figure 5). In pairwise Bonferroni post-hoc comparisons, the (NAA+NAAG)/(Cr+PCr) ratio difference in RPQ (*p* = 0.012) and LPQ (*p* = 0.04) of the LF group was significantly less compared with the HF group (Figure 6).

## 4. Discussion

In this spectroscopy study we investigated the changes in the neurometabolite profile of white matter in RRMS patients who reported fatigue of variable severity. We found significant differences in MRS findings between our three fatigue groups at two time points. More explicitly, we observed a significant reduction in the (NAA+NAAG)/(Cr+PCr) ratio of the right and left frontoparietal WM regions in the HF group compared with the LF group at baseline and year 1. Although the decline in the (NAA+NAAG)/(Cr+PCr) ratio in bilateral posterior quadrants did not reach statistical significance, the (NAA+NAAG)/(Cr+PCr) ratio variation in bilateral posterior quadrants is negative and significantly different in the HF group compared with the LF group. 

As previously mentioned, previous studies have reported biochemical changes limited to specific brain structures or the whole brain [11,12,13,14,15]. To our knowledge, this is the first study investigating changes between two different time points in the neurometabolite profile in MS fatigue, therefore, there are no other studies we can compare our findings to. The MRS grid applied in our study includes frontoparietal and occipitoparietal regions along with the central region of the corpus callosum that encompasses all major WM tracts passing through the periventricular area and may have strong link with lesion load and fatigue pathology in MS. We hypothesized that dissecting the MRS grid into four quadrants will allow relatively fine demarcation of WM areas involved in fatigue and trace associated changes in light of medication use and disease severity. Our findings suggest that the bilteral frontoparietal tracts are more prone to neuronal injury in the HF group. Despite a non-significant variation in overall lesion load and EDSS, the ratio variation between high and low fatigue groups in the posterior regions was significant. Our results are in line with previous cross-sectional findings, adding further evidence on the role of altered white matter neurometabolite profile to the development of MS fatigue.

The FSS scale has been widely used in the clinic to track longitudinal fatigue changes. As we focused only on the physical component of fatigue, we felt that FSS was an appropriate choice. However, the FSS has its shortcomings, such as less precision at higher levels of fatigue than that of the MFIS and the utilization of a 1-week recall period as compared with the 4- week recall period used by the MFIS [33]. Despite previous studies that classify patients into two groups—LH with FSS ≤ 4 and HF with FSS ≥ 5—thus missing the patients with FSS scores between 4 and 5, we included all patients and categorized them into three groups [40]. In our study, despite significant neurometabolite changes in the bilateral frontal WM, the variation in FSS scores was not significant between the two time points. Interestingly, the HF group showed a trend toward a decline in fatigue scores at year 1. This may be explained by the dynamic nature of fatigue that may fluctuate over time, influenced by lifestyle changes, such as sleep patterns, diet, and exercise. Second, given that FSS is a self-report subjective measure of fatigue with several weaknesses, it indicates that more accurate questionnaires with higher sensitivity may be needed to detect subtle changes in fatigue over time. Third, there is a possibility that the neurometabolite changes in the MRS correlate only with a subset of FSS items, something that was not investigated. 

Our analysis encompassed both lesional white matter and NAWM in each quadrant. The contribution of gray matter was minimal, as voxels with gray matter on the boundary of the MRS sequence and voxels with metabolite concentration ratios with a standard deviation higher than 20% were excluded from the analysis. In our study, we calculated the total T2 lesion load, but we did not perform lesional load analysis in each quadrant of the MRS grid. Despite a higher T2 lesion load at year 1 in the HF group compared with MF and LF groups, this was not statistically significant and cannot be accountable for the observed changes. Moreover, given that the T2 lesion load was not statistically different between the two time points and there were no MS relapses recorded, we speculate that the majority of metabolic changes between the three groups, as well as between the baseline and year 1, were attributable to NAWM involvement.

Prior studies showed a correlation between disability and WM metabolic changes [20,21,22]. In our study, all patients had low disability with an EDSS ≤ 4. Despite a higher EDSS in the HF group and total study population at year 1, the increase in EDSS was not statistically significant. In addition, all patients were treated with fingolimod for one year, and its effect on the MRS parameters has not yet been studied. However, prior studies examining the effect of other disease modifying agents, such as natalizumab, showed a yearly increase in total NAA, Cr, and PCr in lesional WM [41]. The administration of laquinimod increased the NAA/Cr, while treatment with a placebo decreased the ratio at 24 months [42]. Therefore, we cannot exclude a beneficial effect of fingolimod on MRS measures, which may cause an attenuation of the changes observed in our study. On the other hand, the relatively short duration of our study may abate the role of fingolimod as a potential source of error in the interpretation of our results.

A key strength of our study is the availability of a unique population of RRMS patients with mild disability that is ethnically diverse, consisting of both African Americans and Caucasians, who have been on a single disease-modifying treatment over the study period. Furthermore, the high test-retest reproducibility of the neurochemical profile measured by the MRS is an additional strength of the study, supportive of existing pathology rather than a technical limitation [43,44]. Our study is not without limitations. We managed to recruit a homogenous group of only RRMS patients with mild disability on a single disease modifying agent, and this resulted in the enrollment of a small sample size that limits further subgroup analysis. In this context, the (NAA+NAAG)/(Cr+PCr) ratio variation in either gender or ethnic group could not be tested because of the small sample size. The lack of a healthy control group limits our ability to draw conclusions regarding causality and does not allow us to distinguish between MS specific or physiological associations. Therefore, it is difficult to draw final conclusions whether or not our findings are attributable solely to MS fatigue, as it is possible that an underlying process could cause fatigue and may contribute to the neurometabolite changes. Despite the limitations discussed above, our robust statistics support our findings, and we investigated for the first time the changes in the neurometabolite profile at two time points. Finally, the unknown effect of fingolimod on the MRS metrics may add to the study limitations. Technical factors, such as the reproducibility of voxel location and other instrument settings in subsequent measurements may also represent potential confounders. 

## 5. Conclusions 

MS patients who are mildly disabled and highly fatigued have a significant decline of the (NAA+NAAG)/(Cr + PCr) ratio in the bilateral frontoparietal WM at baseline and year 1. Our findings may serve as a reference point and warrant further investigation and validation with well-designed, prospective, larger scale studies. 

## Figures and Tables

**Table 1:** Patients’ demographics and MRI characteristics.

RRMS Population	HF Group	MF Group	LF Group	Total	*p*-Value
Number of patients	16	18	14	48	
Gender (M vs. F)	4 vs. 12	4 vs. 14	7 vs. 7	15 vs. 33	0.19
Ethnicity (Cau vs. AA)	5 vs. 11	12 vs. 6	8 vs. 6	25 vs. 23	0.11
Age (years) at baseline	43 ± 2.9	39 ± 3	39 ± 1.7	41 ± 1.7	0.102
Range (years)	(23–55)	(26–45)	(29–47)	(23–55)	
Mean FSS at baseline	6 ± 0.12	4 ± 0.14	1.89 ± 0.2	4.35 ± 0.26	
FSS range	(5.1–7)	(3.1–5)	(1–3)	(1–7)	
Mean FSS at year 1	5.8 ± 0.26	3.81 ± 0.42	2.6 ± 0.46	4.19 ± 1.3	
FSS range	(3.7–7)	(1.1–6.7)	(1–6)	(1–7)	
*p-*value:baseline vs. year 1	0.09	0.1	0.13	0.42	
Median EDSS at baseline	3 ± 0.42	2.71 ± 0.57	2.42 ± 0.55	2.72 ± 0.4	0.754
EDSS range	(1–4)	(1–4)	(1–4)	(1–4)	0.871
Mean EDSS at year 1	3.6 ± 0.49	2.7 ± 0.72	2.35 ± 0.51	3.6 ± 0.34	
EDSS range	(1–4)	(1–4)	(1–4)	(1–4)	
*p*-value:baseline vs. year 1	0.36	0.99	0.92	0.704	
T2 LV (mL) at baseline	14 ± 2.5	18.8 ± 4.8	15.3 ± 5.9	15.6 ± 2.3	0.859
T2 LV range (mL)	(7.4–27.16)	(2.6–40.5)	(1.8–39.7)	(1.8–40.5)	0.64
T2 LV (mL) at year 1	17.5 ± 3	16.2 ± 3.4	15.4 ± 6	18.4 ± 3.8	
T2 LV range (mL)	(7.2–25.5)	(1.9–41.9)	(1–26.4)	(2–39.2)	
*p*-value: baseline vs. year 1	0.75	0.88	0.99	0.76	
Disease duration at baseline	10 ± 1.7	9.2 ± 1.2	8.6 ± 1.9	9.3 ± 1	0.136
Range (years)	(0.5–19.17)	(0.67–14.4)	(0.25–15)	(0.25–19.17)	

Note: MRI—Magnetic Resonance Imaging, RRMS—Relapsing Remitting Multiple Sclerosis, FSS—Fatigue Severity Scale, EDSS—Expanded Disability Status Scale, LV—Lesion volume, HF—High Fatigue, MF—Moderate Fatigue, LF—Low Fatigue, M—Male, F—Female, Cau—Caucasian, AA—African American, mL—milliliter, vs.—versus. The data represents average and standard error of mean along with minimum and maximum values.

**Table 2:** Two-way ANOVA with pairwise comparisons of the NAA+NAAG/Cr+PCr ratio between the fatigue groups.

	NAA+NAAG/Cr+PCr Ratio			
	LAQ	RAQ	LPQ	RPQ
**LF at Baseline**	2.01 ± 0.07	2.08 ± 0.06	2.07 ± 0.05	2.11 ± 0.07
**LF at Year 1**	2.08 ± 0.07	2.07 ± 0.06	2.09 ± 0.05	2.1 ± 0.07
**MF at Baseline**	2.01 ± 0.06	2.08 ± 0.05	2.12 ± 0.06	2.09 ± 0.06
**MF at Year 1**	1.87 ± 0.06	1.9 ± 0.05	2.02 ± 0.06	2.1 ± 0.06
**HF at Baseline**	1.86 ± 0.06	1.89 ± 0.06	2.08 ± 0.05	2.12 ± 0.07
**HF at Year 1**	1.86 ± 0.06	1.91 ± 0.06	1.99 ± 0.05	1.97 ± 0.07
**Fatigue group effect (F(df), *p*-value)**	F(2, 89) = 3.6 *p* = 0.031 *	F(2, 88) = 3.9 *p* = 0.022 *	F(2, 90) = 0.39 *p* = 0.67	F(2, 90) = 0.44 *p* = 0.63
**Time point effect (F(df), *p*-value)**	F(1, 89) = 0.2 *p* = 0.65	F(1, 88) = 1.11 *p* = 0.295	F(1, 90) = 1.84 *p* = 0.17	F(1, 90) = 0.72 *p* = 0.39
Fatigue group * time point effect (F(df), *p*-value)	F(2, 89) = 1.2 *p* = 0.29	F(2, 88) = 1.5 *p* = 0.21	F(2, 90) = 0.71 *p* = 0.49	F(2, 90) = 0.88 *p* = 0.41
**LF vs. MF (Bonferroni post-hoc *p*-value)**	0.3	0.418	0.99	0.99
**LF vs. HF (Bonferroni post-hoc *p*-value)**	0.02 *	0.018 *	0.77	0.76
**MF vs. HF (Bonferroni post-hoc *p*-value)**	0.54	0.333	0.88	0.83

Note: LF—LF group, MF—MF group, HF—HF group, LAQ—left anterior quadrant, RAQ—right anterior quadrant, LPQ—left posterior quadrant, RPQ—right posterior quadrant. Data represents mean ± standard error of mean, *—significant p-value.

**Table 3:** One-way ANOVA of the NAA+NAAG/Cr+PCr ratio difference between the fatigue groups.

	NAA+NAAG/Cr+PCr Ratio Difference			
	LAQ	RAQ	LPQ	RPQ
**Between Baseline and Year 1 in LF**	0.088 ± 0.096	0.0008 ± 0.087	0.087 ± 0.072	0.071 ± 0.052
**Between Baseline and Year 1 in MF**	−0.097 ± 0.077	−0.068 ± 0.07	−0.099 ± 0.058	−0.051 ± 0.042
**Between Baseline and Year 1 in HF**	−0.023 ± 0.082	0.015 ± 0.074	−0.152 ± 0.062	−0.137 ± 0.044
**Fatigue group effect (F(df), *p*-value)**	F(2, 40) = 1.14 *p* = 0.33	F(2, 40) = 0.38 *p* = 0.68	F(2, 40) = 4.69 *p* = 0.041 *	F(2, 40) = 3.41 *p* = 0.015 *
**LF vs. MF (Bonferroni post-hoc *p*-value)**	0.4	0.8	0.14	0.21
**LF vs. HF (Bonferroni post-hoc *p*-value)**	0.9	0.99	0.04 *	0.012 *
**MF vs. HF (Bonferroni post-hoc *p*-value)**	0.9	0.69	0.9	0.5

LF—LF group, MF—MF group, HF—HF group, LAQ—left anterior quadrant, RAQ—right anterior quadrant, LPQ—left posterior quadrant, RPQ—right posterior quadrant. Data represents mean ± standard error of mean, *—significant *p*-value.
